# A machine learning approach to predicting psychosis using semantic density and latent content analysis

**DOI:** 10.1038/s41537-019-0077-9

**Published:** 2019-06-13

**Authors:** Neguine Rezaii, Elaine Walker, Phillip Wolff

**Affiliations:** 1 Department of Neurology, Massachusetts General Hospital, Harvard Medical School, Boston, MA USA; 2 Department of Psychiatry, Emory School of Medicine, Atlanta, GA USA; 3 Department of Psychology, Emory University, Atlanta, GA USA

**Keywords:** Biomarkers, Diseases

## Abstract

Subtle features in people’s everyday language may harbor the signs of future mental illness. Machine learning offers an approach for the rapid and accurate extraction of these signs. Here we investigate two potential linguistic indicators of psychosis in 40 participants of the North American Prodrome Longitudinal Study. We demonstrate how the linguistic marker of semantic density can be obtained using the mathematical method of *vector unpacking*, a technique that decomposes the meaning of a sentence into its core ideas. We also demonstrate how the latent semantic content of an individual’s speech can be extracted by contrasting it with the contents of conversations generated on social media, here 30,000 contributors to Reddit. The results revealed that conversion to psychosis is signaled by low semantic density and talk about voices and sounds. When combined, these two variables were able to predict the conversion with 93% accuracy in the training and 90% accuracy in the holdout datasets. The results point to a larger project in which automated analyses of language are used to forecast a broad range of mental disorders well in advance of their emergence.

## Introduction

Psychotic disorders are among the most debilitating of mental illnesses as they can compromise the most central aspects of an individual’s psychology, their capacities to think and feel. Currently, there are no cures for psychotic disorders, but early detection and intervention may help slow the decline in cognitive functioning.^1–4^ The onset of psychosis is usually preceded by a prodromal phase that is characterized by the expression of subclinical abnormalities in thought, perception, and communication.^2^ The challenge for research is in how to detect the signs of future psychosis while symptoms are still subtle and indistinct. Recent advances in machine learning and natural language processing are making such detection possible. The discovery and amplification of linguistic and behavioral features can be used to construct a *digital phenotype*, a characterization of an individual’s knowledge representations and thought processes that can be used to predict and diagnose the emergence of mental disorders.^5–7^ Here we capitalize on these advances to show how latent features of people’s natural language may be mined to predict the later emergence of psychosis.

The promise of digital phenotyping has been demonstrated in several studies.^8^ Elvevåg et al. observed that cosine similarity between adjacent words produced from a verbal fluency task and sets of words from structured interviews were lower in those with schizophrenia than healthy controls.^9^ Bedi et al. found that average vector similarity between adjacent sentences in free speech, along with several other variables (maximum number of words per phrase and determiners) could be used to identify which clinically high risk (CHR) individuals would convert to psychosis with 100% accuracy.^10^ In a similar set of findings, Corcoran et al. found that semantic coherence in combination with several other variables (maximum coherence, variance in coherence, minimum coherence, and possessive pronouns) could be used to predict the onset of psychosis in two independent groups of CHR individuals.^11^ Mota et al. observed patterns of connectivity between words as measured by graph-theoretical tools could be used to establish the diagnosis of schizophrenia in individuals with first episode psychosis.^12^ Mota et al. also identified sparseness in speech as a variable that discriminates schizophrenia from mania.^13^ The promise of such digital phenotyping in prediction of psychosis highlights the need for additional techniques for objectively measuring other cardinal indicators of psychosis.

One potential candidate for digital phenotyping is *poverty of content*. Poverty of content, or what we will call *low semantic density*, has been commonly acknowledged as a central feature of the language of those with psychosis and predictive of illness outcome.^14–17^ The marker is widely considered a negative symptom,^18^ and as such, may play an especially useful role in the prediction of psychosis as negative symptoms typically occur earlier than positive symptoms during the prodromal phase.^19^ Another core feature of psychosis is *auditory hallucination*.^20–22^ Full auditory hallucination, a positive symptom of psychosis, normally appears relatively late in the course of developing psychosis.^19^ However, given the potentially greater sensitivity of machine learning methods, it may be possible to detect the early signs of auditory hallucination as an increased tendency to implicitly talk about voices and sounds.

In this research, we extend prior work on digital phenotyping by introducing new methods for detecting these two cardinal symptoms of psychosis. Through the technique of *vector unpacking*, we show how semantic density can be measured by partitioning a sentence into component vectors of meaning, which, when divided by the number of words in the sentence, gives a measure of the sentence richness. We also introduce a new computational method for discovering the hidden semantic content of a mental disorder using a method we call *latent content analysis*. This method identifies the latent content of an individual’s speech by comparing it to a large language corpus of language, here 30,000 social media users, making it possible to identify the subtle ways in which the language content of those in the early stages of psychosis stand out from the “norm”.

In investigating digital phenotyping, the predictive ability of several linguistic markers will be analyzed. Semantic density will be compared against two other automated approaches to the measurement of poverty of content: *idea density*, which concerns the number of propositions in a set of words; and *information value*, which concerns the amount of information implied in a vector’s length.^23,24^ We will also compare the predictive ability of semantic density against the related notion of *poverty of speech*, which concerns the amount of language produced by an individual.

## Results

Our findings indicate that during the prodromal phase of psychosis, the emergence of psychosis was predicted by speech with low levels of semantic density and an increased tendency to talk about voices and sounds. When combined, these two indicators of psychosis enabled the prediction of future psychosis with a high level of accuracy.

Speech samples were drawn from 40 participants of the North American Prodrome Longitudinal Study (NAPLS) at Emory University (see Methods). Participants were followed up for 2 years or to the time of conversion. For training the model, we included 30 participants from the second phase of the NAPLS (NAPLS-2). Seven of these individuals converted to psychosis during follow-up (Converters) and 23 did not (Non-converters). For validating the model, we included 10 participants, five Converters and five Non-converters from the third phase of the NAPLS (NAPLS-3). Transcriptions of the recorded Structured Interview for Prodromal Syndromes (SIPS) were used for language analysis. The demographics and clinical information of the participants are shown in Table 1.

**Table 1:** Demographic and clinical information of the participants

	Non-converter	Converter	*p*-value
*Thirty participants of the training set*			
Age	21.496 ± 4.513	20.571 ± 5.623	0.294
Gender (% male)	47.82%	57.14%	0.666
Race			0.554
Caucasian	34.78%	14.29%	
Black	47.82%	57.14%	
Other	17.39%	28.57%	
Estimated IQ	103.363 ± 15.966	97.714 ± 13.124	0.520
Medications			0.109
Antipsychotics	0%	14.29%	
Antidepressants	14.35%	14.29%	
*Ten participants of the validation set*			
Age	22.810 ± 2.347	22.862 ± 3.398	0.978
Gender (% male)	100%	100%	NA
Race			0.533
Caucasian	40%	20%	
Black	20%	20%	
Other	40%	60%	
Estimated IQ	98.60 ± 24.327	98.80 ± 10.426	0.922
Medications			0.549
Antipsychotics	20%	0%	
Antidepressants	40%	40%	

Mean ± standard deviation of age in years and IQ. Also shown are the percentages of male participants, participants under the specified medications, and race. The *p*-values are based on *t*-tests and *χ*^2^ tests between Converters and Non-converters

**Fig. 1:**
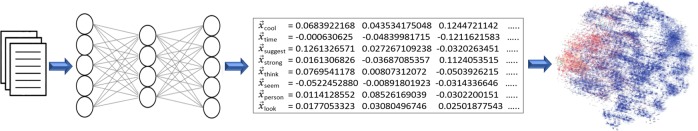
Use of the machine learning technique (Skip-gram) Word2vec to create word embeddings by processing a large body of texts through a two-layer neural network. The weights in the first layer of the network constitute the resulting vectors and specify positions in a high dimensionality space (a word-embedding). A 2-dimensional projection of the 99% most frequent words in English (*N* = 42,234) of this space is shown above (blue = nouns; red = verbs; orange = adjectives; aqua = prepositions)

To perform the vector unpacking method, language samples underwent several pre-processing analyses including lemmatizing the words and tagging them for their part of speech (see methods). To derive sentence meanings, the content words (i.e., nouns, verbs, adjectives, and adverbs) were re-expressed as word embeddings (see Methods). Word embeddings map the words of a language into a vector space of reduced dimensionality. The word embeddings used in this research were generated using the skip-gram version of Word2Vec.^25,26^ The goal of word2vec is to cause words that occur in similar contexts to have similar embeddings. The algorithm can be viewed as instantiating a simple two-layer neural network architecture. In this network, the input layer uses a one-hot encoding method to indicate individual target words. During the feedforward phase, activation travels from the input level to a hidden unit level. Activation from the hidden units travels into a softmax function. The softmax function creates a probability distribution and the system is tuned, using backpropagation, to maximize the probabilities for the words that are being used to train against. The words being trained against code for a word’s context and are specified by a window of words around a target word. In the present research, training was based on 25 years of text from the New York Times (NYT), which includes 42,833,581 sentences. The processing pipeline used to generate word embeddings is shown in Fig. 1.

**Fig. 2:**
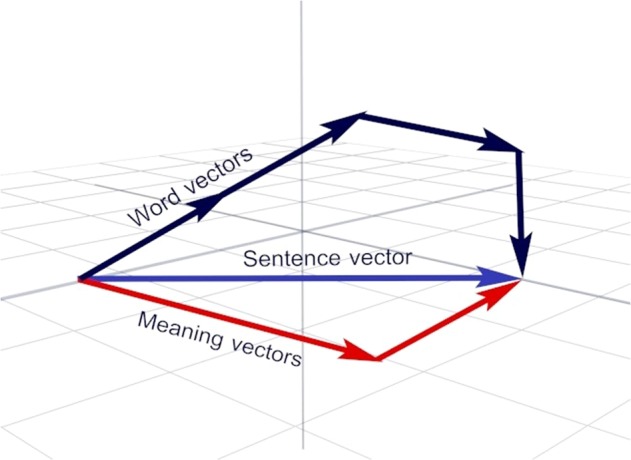
Processes involved in vector unpacking. The word embeddings associated with the words in a sentence (black) when summed produce a resultant sentence vector (blue). Meaning vectors are identified through the learning of weights, which result in a linear combination of vectors that approximates the resultant sentence vector as closely as possible

The meaning of each sentence was derived by summing the vectors (embeddings) associated with each word in the sentence and normalizing by the magnitude of the vectors. A formal specification of these operations is described in the Methods. To determine *semantic density*, the number of meaning components expressed in a sentence must be determined. This was accomplished using a vector decomposition technique called *vector unpacking*. As specified in the Methods, the technique uses gradient descent to discover the linear combination of weighted word vectors (meaning components) that best approximate the observed sentence vectors. When there is minimal semantic overlap among the words in a sentence, all the words in the sentence vector are usually recovered. However, when the semantics of the content words in a sentence overlap in meaning or certain words stand out as unrelated to the semantic emphasis of the sentence, the number of meaning vectors needed to create the sentence is less than the number of content words, resulting in a reduction in semantic density. The process achieved by vector unpacking is depicted in Fig. 2. The word embeddings (black vectors) in a sentence sum to produce a resultant vector for that sentence (blue vector). Vector unpacking finds meaning vectors (red vectors) that, when summed, closely approximate the original sentence vector. In this figure, the number of component vectors (*N* = 2) is less than the number of words (*N* = 4) that were used to create the resultant vector.

Let *S* = {***s***_1_, …, ***s***_*n*_} be the set of sentences in a language sample, indexed by *j*, and |*S*| the number of sentences in that sample. The semantic density of a sentence, *D*_*j*_, was calculated by dividing the number of meaning component vectors, *m*_*j*_, by the number of content words, *n*_*j*_, as specified in the formula

1 $$D_j = \frac{{m_j}}{{n_j}}$$

The mean density of a participant’s language sample, $\bar D$, was computed by summing the semantic densities of the individual sentences in that sample and dividing by the total number of sentences, as specified in the formula

2 $$\bar D = \frac{{\mathop {\sum }\nolimits_j D_j}}{{\left| S \right|}}$$

**Fig. 3:**
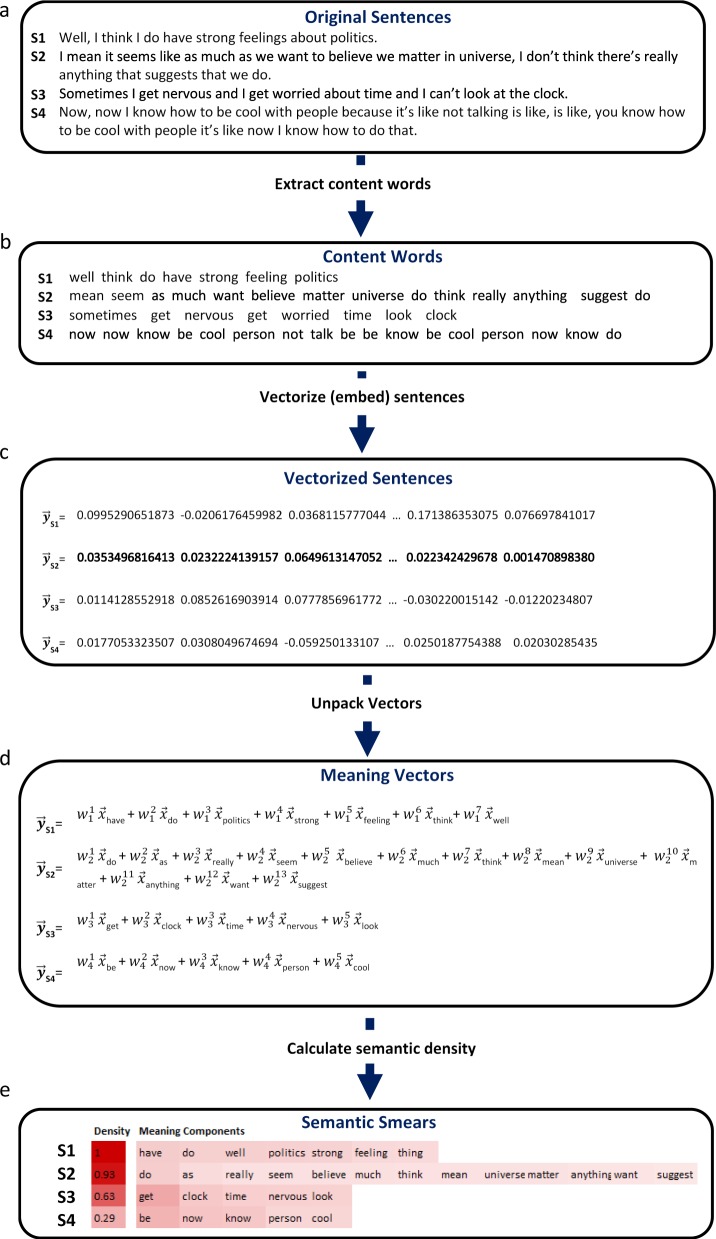
Pipeline used to determine semantic density. **a** Sample sentences of the participants. **b** Original sentences are reduced to their content words (nouns, verbs, adjectives, and adverbs). **c** Word embeddings for each content word are added together to produce a sentence vector. **d** Vector unpacking is used to find the weights that can be used to scale the word vectors so that their addition approximates the sentence vector as closely as possible. **e** The number of meaning component vectors is divided by the number of content words for each sentence to calculate a measure of semantic density. In a semantic smear, the relative weight of the meaning components and final density is specified in the darkness of the surrounding color

The steps involved in deriving this measure of semantic density are summarized in Fig. 3.

### Semantic density as a predictor of conversion

**Fig. 4:**
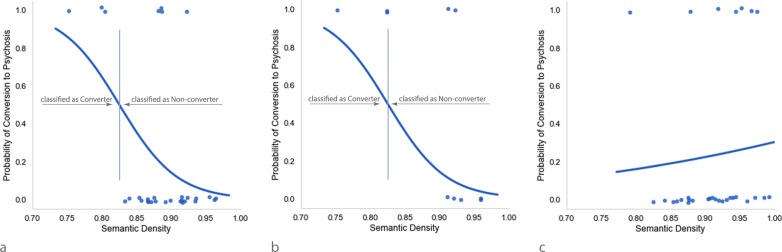
Predicting conversion to psychosis based on semantic density in the original and shuffled samples. Individual points show (with a small amount of jitter) semantic densities of individual participants who either converted to schizophrenia (Probability = 1) or did not (Probability = 0). **a** Probability of conversion to psychosis given semantic density as estimated by binary logistic regression. **b** Probability of conversion to psychosis estimated by a model derived from the training data. **c** Probability of conversion to psychosis given semantic density estimated from randomly shuffling the language samples. After shuffling, conversion to schizophrenia was no longer predicted by semantic density

Given our ability to measure the semantic density of sentences, the language samples of the participants were analyzed to determine whether this aspect of language might predict conversion to psychosis. Regressing SEMANTIC DENSITY on CONVERSION (0 = Non-converter; 1 = Converter), we found that semantic density improved the ability of a model to predict conversion to psychosis, Wald’s χ^2^(1) = 4.401, *p* = 0.036. Figure 4a shows the probability of conversion to psychosis given semantic density as estimated by the logistic regression equation CONVERSION = 19.832 + (−24.022) * SEMANTIC DENSITY. Assuming a probability of conversion cutoff of 0.5, the plot shows that conversion to psychosis was associated with semantic densities of 0.825 or less. A model trained on the training set had an accuracy rate of 86.7% (Precision = 1; F_1_ score = 0.6, Sensitivity/Recall = 0.428, Specificity = 1).

#### Validation of SEMANTIC DENSITY on the holdout dataset

An analysis of the validation dataset confirmed that semantic density is a strong predictor of conversation to psychosis, even for observations not included in the training. When the regression equation fitted to the training dataset was applied to a holdout dataset, conversion to psychosis was predicted with 80% accuracy (Precision = 1; F_1_ score = 0.75, Sensitivity/Recall = 0.60, Specificity = 1). Figure 4b shows the probability of conversion derived from the training dataset and applied to the holdout dataset. As can be seen, the 0.825 semantic density cutoff calculated from the training set resulted in only two misclassifications in the case of the holdout dataset, both involving failures to predict conversion. Figure 4b also shows that if the logistic regression equation had been trained on the holdout dataset alone, the semantic density cutoff would have increased to ~0.88, which would have resulted in a predicted conversion accuracy of 100%.

#### Poverty of content, poverty of speech, part-of-speech, and demographic variables

Interestingly, in the present study of the prodromal phase of psychosis, a poverty of speech effect was not found: the number of content words used by those who converted to psychosis (*M* = 5.10, *SD* = 0.339) was not significantly lower than the number of content words used by those who did not convert (*M* *=* 5.27, *SD* = 0.474), *t*(28) = 0.893, *p* *=* 0.380. The results suggest, then, the best indicator of conversion during the prodromal period may not be poverty of speech, but rather, poverty of content as measured by semantic density. In this study cohort, we did not find any evidence of correlations between semantic density and IQ, *r*(28) = 0.22, *p* *=* 0.239, age, *r*(28) = 0.213, *p* *=* 0.260, or sex, *r*(28) = 0.020, *p* *=* 0.915. In addition, we did not find a significant correlation between semantic density and sentence length, *r*(28) = −.042, *p* *=* 0.822. In our dataset, density of determiners was not a significant predictor of psychosis, but the direction of the effect was consistent with that found in previous studies,^10,11^ Wald’s χ^2^(1) = 2.121, *p* = 0.115.

#### Semantic density as a property of sets of words

Semantic density is measured with respect to specific combinations of words. As such, it should depend on the way the words are grouped together into sentences and not simply on the set of words used in the sample ignoring sentence organization. This prediction was tested by randomly shuffling the content words in the transcripts to disrupt their organization, while keeping all other properties of the text the same. Sentence length and syntax were preserved by switching verbs with verbs, nouns with nouns, and so on. As indicated in Fig. 4c, after the words were randomized, conversion to psychosis was no longer predicted by semantic density, Wald’s χ^2^ (1) = 0.204, *p* = 0.652. This approach is similar to prior work that has used shuffling to establish a baseline level of semantic coherence.^10^ Our results indicate that semantic density is sensitive to the way words are grouped into sentences, and hence with the mental processes used to combine them into sentences.

#### Comparison to alternative approaches to the extraction of semantic density

The technique used to measure poverty of content in this research, vector unpacking, differs from that used in previous research. One such alternative measure is idea density, a quantity that can be measured by dividing the number of verbs, adjectives, adverbs, prepositions, and conjunctions in a sentence by the total number of words.^27,28^ Idea density is calculated automatically in the program CPIDR (www.covingtoninnovations.com/software.html). We analyzed the training dataset in terms of *idea density* using CPIDR 5 and found no evidence Converters (*M* = 0.561, *SD* = 0.026) had lower levels of idea density than Non-converters (*M* = 0.574, *SD* = 0.023), *t*(28) = 1.258, *p* = 0.219. Nor did we find evidence that idea density was related to semantic density, *r*(28) *=* 0.053, *p* = 0.783.

Another approach to the measurement of meaning, *information value*,^23,24^ suggests that the notion of semantic density might be represented in the vector length of a set of words either by calculating the average vector length of a set of words, with vector length being simply the magnitude of a vector (e.g., in the case of the vector[1, 1], vector length would be $\sqrt 2$), or by summing the vectors for a set of words and determining the vector length of their resultant.^24^ After applying vector length analysis to the training dataset, we found no evidence that Converters used words with shorter vectors (*M* = 6.865, *SD* = 0.0318) than Non-converters (*M* = 6.854, *SD* = 0.0301), *t*(28) = 0.839, *p* = 0.409. Nor did we find evidence that the vector length of a resultant vector of a sentence was shorter for Converters (*M* = 3.877, *SD* = 0.534) than Non-converters (*M* = 4.096, *SD* = 0.53612), *t*(28) = 0.944, *p* = 0.353. Lastly, we observed no relationship between semantic density and either average vector length, *r*(28) = −.106, *p* *=* 0.576, or sentence resultant length, *r*(28) = −.089, *p* = 0.641. The lack of any association with semantic density should not be interpreted as implying that vector length is semantically inert. We found that vector lengths correlated negatively with word frequencies, *r* = −.132, *p* < 0.0001, and positively with the number of content words summed to create a sentence vector, *r*(70) = 0.414, *p* = < 0.001. It is entirely possible that the notions of idea density and information value might capture psychologically interesting dimensions of language, but our analyses suggest that these notions do not capture the same information as semantic density as measured by vector unpacking and are not predictive of conversion to psychosis.

#### Machine and human ratings of semantic density

The results of a simple validation experiment confirm that the notion of semantic density measured by machine learning resembles the subjective notion of semantic density as understood by humans. In this experiments, human participants (*N* = 30) rated 72 sentences produced by the participants in the training sample. The machine rating of semantic density was correlated with that of human raters, *r*(70) = .42, *p* = < 001. While only moderate in strength, the degree of correlation between human raters and the vector unpacking algorithm was far better than between human raters and other automated measures of semantic density. The correlation between idea density, as measured by CPIDR5,^27^ and human ratings of semantic density was, in fact, in the opposite direction of what was expected, *r*(70) = −.199, *p* *=* 0.093, and the correlation between information value, as measured by vector length,^24^ showed no relation to human judgments of semantic density, *r*(70) = 0.061, *p* = 0.613. Thus, while several measures of semantic density have been proposed in the literature, only vector unpacking generates values related to those of human raters. We further note that in past research, the inter-rater reliability of human judgments of ideational richness has tended to be relatively low,^14,29^ suggesting that such judgments are difficult for human judges, which might account for the moderate strength of the association between human raters and vector unpacking.

### Latent content as a predictor of conversion

The symptoms of full psychosis may not only involve the lack of certain features—as reflected in absence of certain kinds of content—but also the presence of linguistic content not typical observed in the speech of healthy individuals. While negative symptoms tend to precede positive symptoms,^2,19^ the early signs of positive symptoms might nevertheless begin to appear in the content of language during the prodromal period.

Such content can be discovered using a set of techniques we call *Latent Content Analysis* (see Methods). The first step in this analysis involves re-representing the participants’ sentences as vectors. This was accomplished by summing the word embeddings associated with the content words of each sentence and normalizing them to a vector length of 1. To identify latent semantic contents, we selected the 95% most commonly written words in English as reflected in word frequencies in the New York Times corpus (*N* = 13,592) and used them as semantic probes. This was accomplished by re-expressing the probe words as word embeddings, calculating the cosine between each probe word and each participant’s sentence, and retaining the highest cosine for each word across the sentences for each participant. Importantly, the method allows for the discovery of words that were never actually used by the participants; hence, the technique can be used to discover latent meanings. To obtain semantic themes across participants, the cosines to all 13,592 probe words were averaged across the participants in the Converter and Non-converter groups. The next step in Latent Content Analysis weighs words for their informativity. Finding informative words requires identifying the word meanings used more often than normal. This can be accomplished by determining each probe word’s base-rate cosine, that is, the degree to which the word is similar to the meaning of sentences found in an average conversation. This was achieved by constructing a corpus of 30,000 individuals who engaged in online conversations on the social media platform Reddit. The corpus was roughly 401 million words in size, making it large enough to establish base-rate cosines. Average cosines were obtained by comparing the 13,592 probe words with the sentences in this Reddit corpus. Once obtained, the average cosines to the 13,592 probe words for the Reddit corpus could be combined with those associated with the Converters and Non-converters to form two 13,592 × 2 (probe word × group) matrices, one for the Converters and the other for the Non-converters. Distinctive content words were identified using the tf-idf (term frequency-inverse document frequency) weighting algorithm,^30^ which is a method that weighs the values in a matrix to better specify their diagnostic importance. The algorithm addresses the problem of large cosines due to high frequencies by factoring in the effect of base-rates. High cosine values are retained so long as they are high for one group and not another. The 50 probe words with the largest positive cosines after tf-idf were retained for further analysis.

It was anticipated that the top probe words might form clusters of meaning. This possibility was investigated by re-expressing the top 50 probe words using the NYT word embeddings described earlier. The dimensionality of the word embeddings was reduced from 200 to 2 dimensions to remove noise and accentuate the most important semantic dimensions using the t-SNE learning algorithm.^31^ Clusters were identified by applying the k-means++ cluster algorithm, which separates elements into groups by minimizing within-cluster sum-of-squares. The number of clusters was determined by running the algorithm for different numbers of k and choosing the k that maximized the Silhouette Coefficient.^32^

**Fig. 5:**
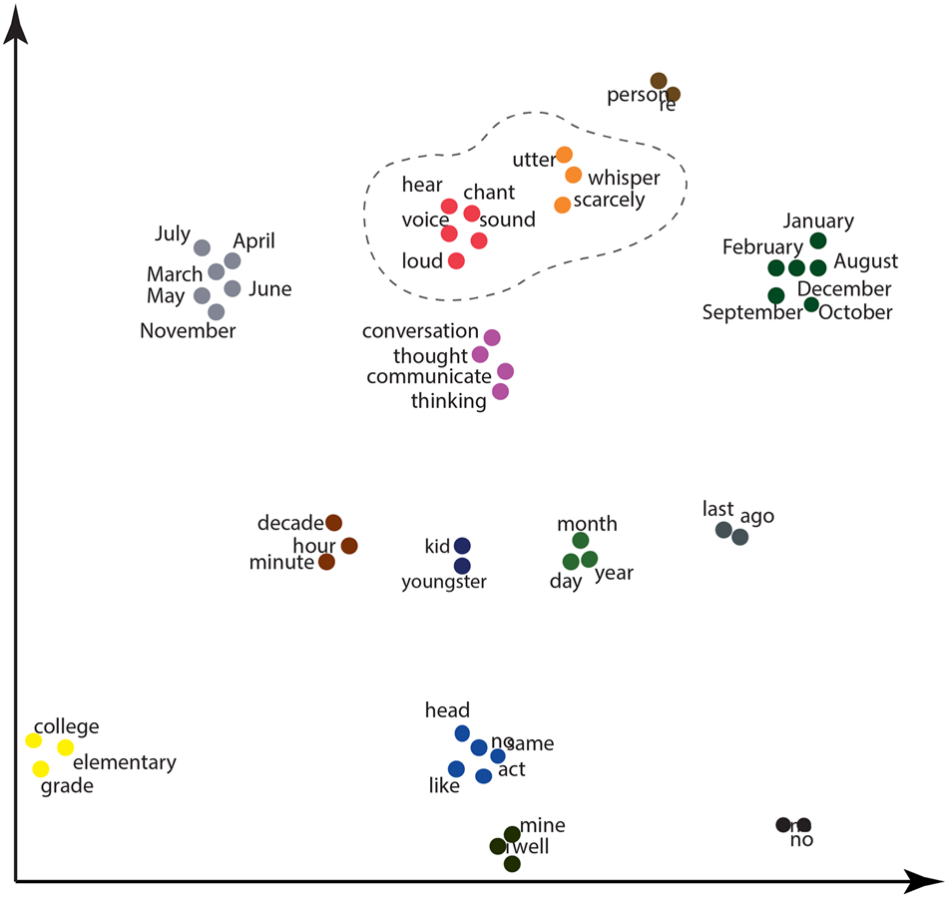
Text plot of words that distinguished the language of the Converters from the language of 30,000 Reddit users. Word positions were determined after dimensionality reduction of the word embeddings and clustering the positions using k-means++. The encircled clusters concern concepts related to voices and sounds

Figure 5 shows the semantic clusters formed out of the probe words that distinguished the language of the Converters from the 30,000 Reddit users. As can be seen, the top 50 probe words fell into 14 semantic clusters. Some of the resulting clusters such as ‘yes/no’ directly reflect the structured interview context from which the language samples were collected. However, several of the clusters indicate topics of potential diagnostic value. Most notably, the language of the Converters tended to emphasize the topic of auditory perception, with one cluster consisting of the probe words *voice*, *hear*, *sound*, *loud*, and *chant* and the other, of the words *whisper*, *utter*, and *scarcely*. Interestingly, many of the words included in these clusters–like the word *whisper*–were never explicitly used by the Converters but were implied by the overall meaning of their sentences. Such words could be found because the cosines were based on comparisons between probe words and sentence vectors, not individual words. Although the Non-converters were asked the same questions, their responses did not give rise to semantic clusters about voices and sounds.

Given their clear connection to auditory hallucination, it is possible that the probe words referring to voices and sounds might not only distinguish Converters from Reddit users, but also Converters from Non-converters. To test this possibility, the cluster based on *voice*, *sound*, *hear*, *chant*, and *loud* was converted into a predictor variable. This was achieved by summing the word embeddings associated with these probe words, normalizing by the magnitude of the vectors, and obtaining the cosine between this cluster vector and all of the sentence vectors from the Converter and Non-convert groups. A VOICES predictor variable was constructed by selecting the largest cosine between the cluster vector and the sentence vectors of each participant.

**Fig. 6:**
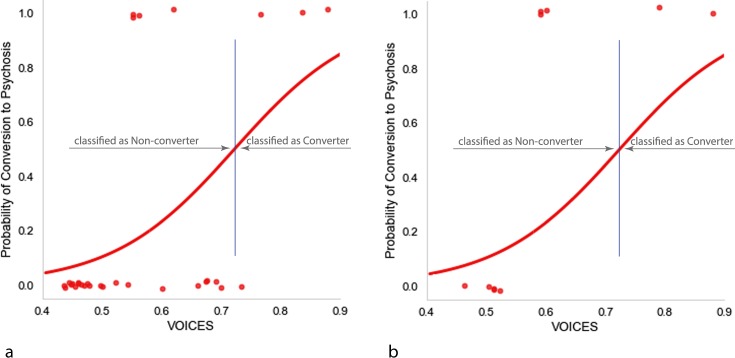
Probability of conversion to psychosis given VOICES. **a** Prediction over the training set. **b** Probability of conversion using VOICES based on training dataset applied to data from the holdout dataset

Regressing VOICES on CONVERSION indicated that talk about voices and sounds improved the ability of a model to predict conversion to psychosis, Wald’s χ^2^(1) = 5.546, *p* = 0.019. Figure 6a shows the probability of conversion to psychosis given the logistic regression equation CONVERSION = −7.047 + (9.744) * VOICES. A model trained on the training set had an accuracy rate of 83.3% (Precision = 0.75; F_1_ score = 0.55, Sensitivity/Recall = 0.428, Specificity = 0.956). Assuming a probability of conversion cutoff of 0.5, the plot shows that conversion was associated with cosine similarities to VOICES that were greater than 0.742. Interestingly, as also shown in Fig. 6b, had VOICES been regressed on CONVERSION using the holdout data alone, prediction accuracy would have been 100%.

#### Validation of VOICES on the holdout dataset

An analysis of the holdout data confirmed that VOICES remained a strong predictor of conversion even on unseen data. When the regression fitted to the training data was applied to the holdout data, conversion to psychosis on the basis of VOICE could be predicted with 70% accuracy (Precision = 1; F_1_ score = 0.571, Sensitivity/Recall = 0.40, Specificity = 1). As can be seen in Fig. 6b, the 0.742 cutoff calculated from the training set resulted in three false negative errors in the holdout dataset.

The language samples used in these analyses were drawn from structured interviews. A potential concern is that the effect of voices and sounds may have been more prominent in the Converters than Non-converters due to the structure interview format. In order to test this possibility, we first analyzed the speech of the interviewers in the same way we analyzed the speech of the participants. A model in which the VOICES vector was tested against sentences generated by interviewers was not predictive of conversion, Wald’s χ^2^(1) = 2.247, *p* = 0.134, implying that the tendency to talk about voices was not directly induced by the language of the interviewers. We also examined the possibility that the Converters might have been asked more questions about voices and sounds than Nonconverters because the Converters had endorsed perceptual changes. We tested this possibility by analyzing the P4 subscale of the SIPS interview, which contains six questions focusing on auditory distortion, illusion, and hallucination. When a participant endorses experiencing perceptual changes, their P4 scores are increased. We found, however, that P4 scores for Converters (*M* *=* 1.20, *SD* *=* 2.19) were effectively the same as for Nonconverters (*M* *=* 1.19, *SD* *=* 1.26), *t*(28) = 0.013, *p* = 0.989. In sum, two sources of evidence argue strongly against the effect of VOICE being due to the structure interview.

#### Language samples indicating early stages of change in auditory perception

The references to voices and sounds in our data nicely demonstrate prior observations made in literature. Crucially, the way prodromal participants seem to experience voices and sound differs from those in patients with overt psychosis. In the early stages of auditory hallucination, individuals realize that there is something wrong with their perceptual experience^33^ and that their thoughts and perception are somewhat mixed.^34,35^ As auditory hallucinations become fully formed, patients with overt psychosis report hearing multiple distinct voices other than their own.^36^ The following excerpts from two of the converters exemplify statements illustrative of the early stage of auditory hallucination.

Patient 1) “…You know I talk to myself but I don’t … I don’t know if it is me. I mean if I talk to myself in the mirror you know. I’m talking to me. But how can I have a conversation with myself? I say stuff in my head as if I am talking to me and it’s funny and I laugh like I didn’t know that I was going to say that…”

Patient 2) “I would hear something that sound like a plane engine or like a really… you know… a really far off motor. It never went away entirely. It’s gone a lot more in the past couple of months since Christmas. It just sounds like that… it sounds like a little flame or a cellular… a digital motor.”

### A predictive model based on SEMANTIC DENSITY and VOICES

**Fig. 7:**
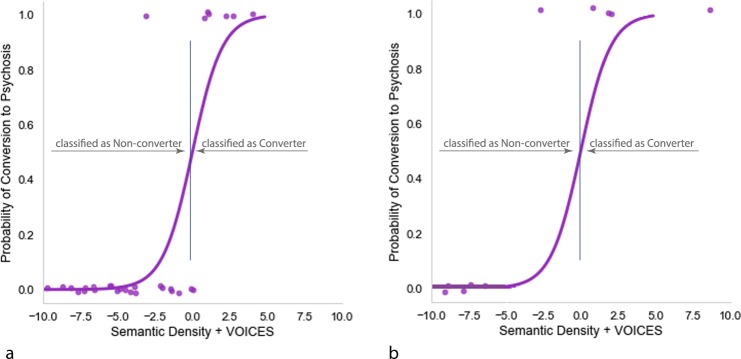
Probability of conversion to psychosis based on SEMANTIC DENSITY and VOICES. **a** Prediction over the training set. **b** Probability of conversion based on training dataset applied to data from the holdout dataset

When SEMANTIC DENSITY and VOICES are combined, the resulting model predicts the emergence of psychosis with 93% accuracy (Precision = 0.86; F_1_ score = 0.86, Sensitivity/Recall = 0.86, Specificity = 0.96). Both SEMANTIC DENSITY, Wald’s χ^2^(1) = 4.047, *p* = 0.044, and VOICES, Wald’s χ^2^(1) = 5.323, *p* = 0.021, contributed to the model’s predictive performance. Figure 7a shows the probability of conversion to schizophrenia for the logistic regression equation CONVERSION = 35.828 + (−57.254) * SEMANTIC DENSITY + (20.483) * VOICES. In this model, all but one of the seven convertors are above the 0.5 probability cutoff.

#### Validation of SEMANTIC DENSITY and VOICES on the holdout dataset

When the regression equation fitted to the training data was applied to the holdout data, it resulted in 90% prediction accuracy (Precision = 1; F_1_ score = 0.89, Sensitivity/Recall = 0.80, Specificity = 1). As shown Fig. 7b, all but one of the converters to psychosis had probabilities greater than the 0.5 probability cutoff of 0.0. Also as shown in Fig. 7b, had the model been based on the holdout data alone, regressing SEMANTIC DENSITY and VOICES on CONVERSION would have allowed for 100% prediction accuracy.

#### Association between computational linguistic features and clinically rated symptoms

**Fig. 8:**
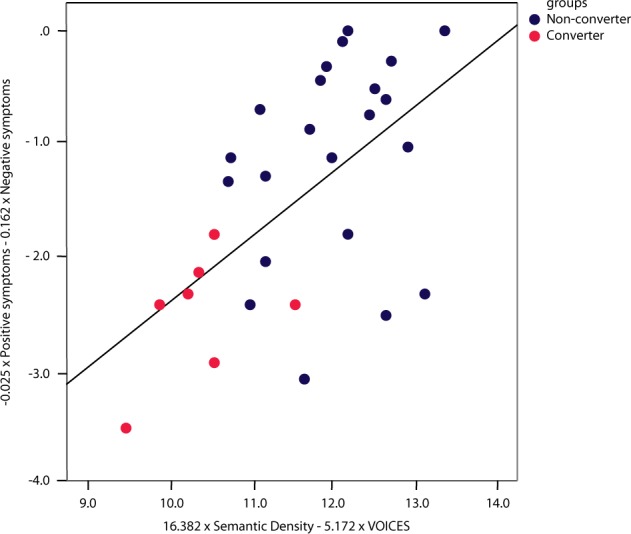
Scatterplot between Semantic density + Voices on the *X*-axis and positive + negative symptoms on the *Y*-axis. Canonical correlations indicated that the linguistic indicators are related to classic variables on standardized rating scales (*r* = 0.568, *p* = 0.012)

Combining semantic density and VOICE in a single model improved prediction performance in part because the two variables capture different kinds of information, as reflected in the lack of correlation between them, *r*(28) = 0.069, *p* *=* 0.717. Prior research suggests that semantic density should align with negative symptoms, and VOICE with positive symptoms.^18^ We investigated this possibility using the negative and positive scores on the SIPS obtained within 6 months of the interview. As predicted, negative symptoms correlated negatively with semantic density, *r*(28) = −.446, *p* *=* 0.013, but not VOICE, *r*(28) = 0.316, *p* = 0.089., and positive symptoms correlated positively with VOICE, *r*(28) = 0.411, *p* = 0.024, but not semantic density, *r*(28) = −.134, *p* = 0.480. The pattern observed in these individual correlations was further supported by canonical correlations, which indicated a latent variable associated with semantic density and VOICE correlated positively with the latent variable associated with negative and positive symptoms, *r* = 0.568, *p* = 0.012. The positive relation between these variables is reflected in the scatterplot shown in Fig. 8. The correlation implies that the semantic variables extracted from text are related to classic variables on standardized rating scales. Crucially, however, when positive and negative symptoms are combined, the resulting model predicts the emergence of psychosis with only 80% accuracy (Precision = 0.66; F_1_ score = 0.4, Sensitivity/Recall = 0.286, Specificity = 0.956). Thus, a predictive model based on linguistic features outperforms one using standardized clinical ratings.

#### Appropriateness of the New York Times corpus

Semantic density and latent content analysis use word embeddings. In the above analyses, the word embeddings were based on 25 years of the New York Times. The New York Times offers a large amount of sophisticated examples of English language usage but differs from the kind of language typically used by the participants in our study. It remains an open question whether the results would have differed, if not improved, had the corpus been more representative of the kind of language used by our participants. To investigate this possibility, a new corpus was created of comparable size to the NYT corpus based on 2 weeks of posts to the social media platform Reddit. The corpus contained 61,866,211 sentences and 1.1 billion words and was processed in the same way as New York Times corpus. We subsequently repeated the analyses conducted with the NYT corpus, but this time with word embeddings produced from Reddit. The results were nearly the same as before. Prediction accuracy using semantic density resulted in 87% accuracy for the training dataset and 80% accuracy for the holdout dataset using the training model, and 100% accuracy when the holdout dataset was analyzed on its own. Prediction accuracy using latent semantic content was 87% for the training dataset and 60% on the holdout dataset when using the training model, but 100% accurate when the holdout dataset was analyzed on its own. When the two measures were combined, the accuracy was 90% for the training dataset and 90% for the holdout dataset (Precision = 1; F_1_ score = 0.89, Sensitivity/Recall = 0.80, Specificity = 1). The results replicate the findings from the previous analyses, indicating that the effects associated with semantic density and latent content are largely independent of the corpus.

## Discussion

This work is a proof of concept study demonstrating that indicators of future mental health can be extracted from people’s natural language using computational methods. Our approach included the development of a mathematical algorithm for unpacking the meaning components of a sentence as well as a computational pipeline for identifying the kinds of thought content that are potentially diagnostic of mental illness. Finally, we showed how the linguistic indicators of mental health, semantic density and talk about voices, could predict the onset of psychosis at high levels of accuracy.

In addition to predicting the onset of psychosis, the methods provide insight into the thought processes affected in the emergence of psychosis. Our results from randomly shuffling words showed that semantic density was a function of the way words were organized into sentences, not simply which words were used across sentences. The results are thus consistent with work suggesting that patients with psychosis have impairments in the integration of words to generate higher order meaning.^37^ These results thus extend the literature on how computational methods can be used to predict and diagnose psychosis from speech. In an analysis of the speech, Mota et al.^13^ could distinguish those with mania from schizophrenia by drawing on a set of ten different graph measures with 93.7% accuracy.^13^ Also based on the graph analyses of thought disorganization, Mota et al.^12^ found that connectedness of language was lower in schizophrenia patients than in healthy control participants 6 months in advance of the schizophrenia patients receiving a diagnosis, allowing prediction accuracy of 91.7%.^12^ Elvevåg B, et al.^9^ differentiated schizophrenia from healthy controls using semantic coherence based on average cosine similarity of words.^9^ This method was extended by Bedi et al.,^10^ who found that the combination of semantic coherence, the number of determiner pronouns and maximum phrase length, could predict conversion to schizophrenia in the training set with 100% accuracy. Lastly, Corcoran et al.^11^ found that four predictor variables in free speech—maximum coherence, variance coherence, minimum coherence, and possessive pronouns—could be used to predict the onset of psychosis with 83% accuracy. In addition to measuring abnormal thought processes, the current study offers a method for the early detection of abnormal auditory experiences at a time when such abnormalities are likely to be missed by clinicians.

This study had a relatively small number of participants. A well-known problem in these studies is overfitting and consequently poor generalization.^38^ In the current study, we took steps to guard against overfitting by limiting the number of predictive variables and testing our results against a holdout set of data. While a low number of predictor variables is desirable, generalization performance can also be compromised by too few variables. In the current study, we found that the linguistic variables of semantic density and talk about voices produced the best fit to the data. Semantic density was found to correlate with negative symptoms and talk about voices with positive symptoms. Successful prediction of psychosis may depend, then, on models that including at least two kinds of variables: those associated with positive and negative symptoms.

In future studies, larger cohorts of patients, more variety in the neuropsychiatric disorders under investigation, and the inclusion of healthy controls could help clarify the generalizability and reliability of the results. Further research could also investigate the ways in which machine learning can extract and magnify the signs of mental illness. Such efforts could lead to not only an earlier detection of mental illness, but also a deeper understanding of the mechanism by which these disorders are caused.

## Methods

### Participants

This study protocol was approved by the Institutional Review Board of Emory University. This study included 40 participants of the North American Prodrome Longitudinal Study (NAPLS) at Emory University. All participants provided written informed consent to participate in this study. 30 participants were included from the second phase of the study (NAPLS-2) for training the prediction model. The remaining 10 participants were included from the third phase of the study (NAPLS-3) for testing the model. All participants were native English speakers who consented to video-recording their baseline diagnostic interviews. Although NAPLS-2 consists of a large sample size, audio/video recordings were not available on all participants. In this study, we included seven individuals who converted to psychosis within the 2-year follow-up and had video recordings available. We then included 23 Non-converters participants who demographically matched the Converters. As described in detail in a previous study,^39^ exclusion criteria for all groups included previous diagnosis of a psychotic disorder, a history of substance dependence, a neurological disorder, or an estimated IQ < 70.

### Protocol

Detailed descriptions of the recruitment, participants and procedures of NAPLS are provided in previous published reports.^40^ Participants were selected for this study if they had agreed to recording the diagnostic interview, the audio recording was of sufficient quality for transcription, and follow-up data were available to determine diagnostic outcome. Participants were evaluated using the Structured Interview for Prodromal Syndromes (SIPS)^41^ and the Structured Clinical Interview for DSM-IV Axis I Disorders^42^ by trained interviewers who met high reliability standards.^43,44^ Participants were followed up at 6-month intervals to the time of conversion to psychosis or to the last contact at two years after baseline assessment. Conversion to psychosis was determined by SIPS criteria of a rating of ‘6’ on any of the positive psychotic symptoms assessed with the instrument, which represents the threshold severity for clinical delusions, hallucinations, paranoia, or thought/communication disorder. This severity level corresponds to the level of severity required for a DSM-IV diagnosis of a psychotic disorder.

### Speech analyses

Video recordings of SIPS interviews for all the 40 participants were transcribed by the same research member, who was blind to the conversion status. Semantic density and content analyses started with a series of pre-processing stages. First, speech from participants was separated from speech produced by interviewers. Second, individual sentences and part-of-speech (POS) categories were identified. This was accomplished using the Stanford Probabilistic Context-Free Grammar (PCFG) parser,^45^ with the maximum length of the sentence set to 60 words. In addition to applying POS tags to individual words (e.g., nouns, verbs, adjectives, adverbs, determiners, and pronouns), the Stanford Parser was able to tokenize sentences, that is, automatically identify all the sentences in a string of text. Third, to focus the representations on the main meanings of the text, the sentences of both the participants and interviewers were reduced to just the content words, that is, the nouns, verbs, adjectives, and adverbs of the sentence.^46^ Fourth, to facilitate generalization, the words were expressed in their uninflected forms through a process called lemmatization. For example, words such as jumped, jumps, jumping were all expressed as the word *jump*. Lemmatization was achieved using the Natural Language Toolkit’s (NLTK) WordNetLemmatizer module.

### Statistical analyses

To investigate the potential differences between converters and nonconverters we used independent-samples t-tests, *t*. To examine associations between semantic density and other measures of semantic richness, as well as, between linguistic features and negative and positive symptoms, we used Pearson correlation coefficient, *r*.

### Vectorizing (embedding) words using Word2vec

The current project used the skip-gram version of Word2vec available in the Python module Gensim.^47^ The context window was five words before and after the target word. The number of hidden units was 200. The network was trained on 25 years of text from the New York Times (42,833,581 sentences) created by the Linguistic Data Consortium.^48^ Before training, POS tags were attached to the lemmatized form of each word in the corpus to improve generalization. The network iterated through the corpus four times during training. The quality of the word embeddings produced by Word2Vec has been shown to outperform other embedding methods, such as Latent Semantic Analysis (LSA) when the training corpus is large (e.g., greater than 1 million words^49^).

### Sentence vectors

Sentence vectors were derived by summing the word embeddings associated with the words in each sentence and normalizing by the magnitude of the resultant. For example, given the sentence *Yesterday I heard a voice*, the meaning of the sentence was specified by adding the word embeddings associated with NN_*yesterday*, VB_*hear*, and *NN_voice*, and scaling the result by 1/∥resultant∥. Formally, let ***X*** = {***x***_1_, …, ***x***_*n*_} be the set of normalized word vectors computed from a word embeddings method, such as Word2vec. Let *I*_*j*_ = {*k*^1^, …, *k*^*n*^} be a set of indexes for the word vectors associated with the words of a particular sentence, *y*_*j*_, and let ***Y*** = {***y***_1_, …, ***y***_|*S*|_} be the set of sentence vectors that are normalized by dividing the sum of the associated word vectors in the sentence by their magnitude, as specified in (3):

3 $${\boldsymbol{y}}_j = \frac{{\mathop {\sum }\nolimits_{k \in I_j} {\boldsymbol{x}}_k}}{{\parallel \mathop {\sum }\nolimits_{k \in I_j} {\boldsymbol{x}}_k\parallel }}$$

### Vector unpacking

Measuring semantic density first involved determining the number of meaning components, ***x***_*i*_, in a sentence. A meaning component is an idea or set of ideas labeled by a word and associated with a word vector. The number of meaning components used to express the ideas in a sentence often corresponds to the number of words in that sentence, but not always. A sentence with the phrase *canine dog*, for example, might require only one word vector component to specify the meaning of this phrase because the two words in the phrase are so highly similar in meaning.

Vector unpacking involves assigning weights, *w*_*ij*_, (*i* = word; *j* = sentence) that scale a corresponding meaning vector, ***x***_*i*_, and using the linear combination of these weighted meaning vectors to estimate a sentence vector, ${\hat{\boldsymbol y}}_j$, as expressed in

4 $${\hat{\boldsymbol y}}_j = \mathop {\sum }\limits_i w_{ij}{\boldsymbol{x}}_i$$

To measure the discrepancy between the actual sentence vector ***y***_*j*_, and the estimated ${\hat{\boldsymbol y}}_j$ sentence vector we used a Euclidian cost function,

5 $$E = \frac{1}{2}\mathop {\sum }\limits_j ({\boldsymbol{y}}_j - {\hat{\boldsymbol y}}_j)^2$$

To determine how to minimize the cost function through changes in the weights, we calculated the partial derivative of the cost function with respect to weights using the chain rule.

6 $$\frac{{\partial E}}{{\partial w_{ij}}} = \frac{{\partial E}}{{\partial \left( {{\boldsymbol{y}}_{\boldsymbol{j}} - {\hat{\boldsymbol y}}_{\boldsymbol{j}}} \right)}} \cdot \frac{{\partial \left( {{\boldsymbol{y}}_{\boldsymbol{j}} - {\hat{\boldsymbol y}}_{\boldsymbol{j}}} \right)}}{{\partial w_{ij}}}$$

Differentiating Eq. (6), changes in $\left( {{\boldsymbol{y}}_{\boldsymbol{j}} - {\hat{\boldsymbol y}}_{\boldsymbol{j}}} \right)$ with respect to *w*_*ij*_ are given by the equations in (7):

7 $$\frac{{\partial E}}{{\partial w_{ij}}} = \frac{{\partial E}}{{\partial ({\boldsymbol{y}}_{\boldsymbol{j}} - {\hat{\boldsymbol y}}_{\boldsymbol{j}})}} \cdot \frac{\partial }{{\partial w_{ij}}}\left( {{\boldsymbol{y}}_j - \mathop {\sum}\limits_i {w_{ij}{\boldsymbol{x}}_i} } \right) = \frac{{\partial E}}{{\partial ({\boldsymbol{y}}_{\boldsymbol{j}} - {\hat{\boldsymbol y}}_{\boldsymbol{j}})}} \cdot ( - {\boldsymbol{x}}_{\boldsymbol{i}})$$

Changes in *E* with respect to $\left( {{\boldsymbol{y}}_{\boldsymbol{j}} - {\hat{\boldsymbol y}}_{\boldsymbol{j}}} \right)$ are given by equations in (8).

8 $$\frac{{\partial E}}{{\partial \left( {{\boldsymbol{y}}_{\boldsymbol{j}} - {\hat{\boldsymbol y}}_{\boldsymbol{j}}} \right)}} = \frac{\partial }{{\partial \left( {{\boldsymbol{y}}_{\boldsymbol{j}} - {\hat{\boldsymbol y}}_{\boldsymbol{j}}} \right)}} \cdot \frac{1}{2}{\sum} {({\boldsymbol{y}}_j - {\hat{\boldsymbol y}}_j)^2} = {\sum} {({\boldsymbol{y}}_j - {\hat{\boldsymbol y}}_j)}$$

Putting the equations together, Eq. (9) expresses how changes in the cost function are related to changes in the weights.

9 $$\frac{{\partial E}}{{\partial w_{ij}}} = - {\sum} {({\boldsymbol{y}}_j - {\hat{\boldsymbol y}}_j)} \cdot {\boldsymbol{x}}_i$$

**Fig. 9:**
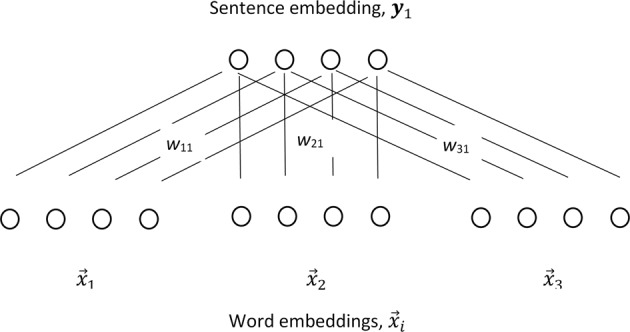
Neural network that learns how to weigh word embeddings. The weights are learned so that when they are summed, they approximate a particular sentence embedding

The process of minimizing the sum of squared errors can be implemented in an artificial neural network like the one in Fig. 9.

In the network in Fig. 9, the input level consists of all of the word embeddings in the lexicon, ***x***_1_, …, ***x***_*n*_. The output level are sentence vectors, ***y***_*j*_. Note that the network is not fully connected, that is not every unit in the input layer is connected to every unit in the output level. Rather, as depicted in Fig. 9, the first element of each word-embedding vector in the input level connects to the first element of the sentence embedding vector in the output level, the second element to the second element, and so on. Moreover, all of the links from each word-embedding to the sentence embedding share a common weight. The task of such a network is to find a set of weights that scale each word-embedding so that when all of the word embeddings in the input layer are summed, they approximate the sentence embedding vector as closely as possible.

The gradient calculated in Eq. (9) can be used to determine the change in weights that minimize the discrepancy between the actual sentence vectors and the estimated sentence vectors, as specified in Eq. (10).

10 $$\Delta {\boldsymbol{w}} = \eta \partial E/\partial {\boldsymbol{w}}$$

The learning rate in Eq. (10), *η*, reduces the gradient slightly so that the gradient descent occurs smoothly. Because the cost function is computed over vector quantities, the partial derivatives associated with each unit in the output layer are summed to modify the single weight that scales an entire word-embedding.

11 $$\partial E/\partial w_{ij} = {\mathrm{sum}}\left\{ { - \mathop {\sum} \limits_i ({\boldsymbol{y}}_j - {\hat{\boldsymbol y}}_j){\boldsymbol{x}}_i} \right\}$$

The weights can be updated by adding the gradient to the existing weights:

12 $$w_i: = w_i + \Delta w_i$$

During training, if the weights fall below a threshold, they are set to 0. The threshold is given by

13 $$\frac{{{\mathrm{iteration}}\,{\mathrm{number}}}}{{\tau \ast {\mathrm{max}}\left\{ {{\mathrm{iterations}}} \right\}}}$$

In the studies reported in this paper, *τ* = 100 and max{*iterations*} = 5000. This cutoff function effectively prunes the number of word embeddings to a relatively small number of highly influential word embeddings. In practice, this pruning results in 30–50 non-zero weights, thus giving rise to far more non-zero weighted word embeddings than words in the typical sentence. Interestingly; however, the weights associated with sentence embeddings show a pattern in which several of the word embeddings have especially high weights. In high-density sentences, the word embeddings with high weights are nearly always the same words that appeared in the original sentence. Given this pattern, all analyses in this paper were based on word embeddings having high weights. Word embeddings with high weights were selected by first rank ordering the weights and then iteratively partitioning the sequence of weights into two groups. For each partitioning of the weights, within and between variances of the weights were calculated and used to compute an F ratio. The partitioning of weights resulting in the highest F ratio was used to select the word embeddings with the highest weights. For sentences in which the semantic density was less than or equal to 0.25, partitioning was based on the second highest F ratio because when semantic densities were this low, the partitioning was usually due to especially high numbers of word repetitions.

### Alternative to semantic density

One possible alternative to semantic density is information value, which is based on the average vector length of a word. To test whether information value was potentially predictive of psychosis, vector lengths were obtained using a version of Word2vec written to work with Tensorflow available at https://github.com/tensorflow/tensorflow/blob/master/tensorflow/examples/tutorials/word2vec/. Access to the code made it possible to save the word embeddings without normalization. The embeddings were trained on the Text8Corpus available at https://rare-technologies.com/deep-learning-with-word2vec-and-gensim/.

### Latent content analysis

The goal in Latent Content Analysis is to identify the semantic content that distinguishes a body of text from a baseline body of text. The baseline body of text can be used to establish what is normal or typical. By contrasting the smaller body of text with the larger body of text, the unique aspects of the smaller body of text can be made more obvious and accentuated. Without such a comparison, a content analysis of a small body of text would contain information not only about what is unique to that text, but also information about what is common to other texts. Text from the social media site Reddit was used to construct a corpus reflecting the content of normal conversations. On Reddit, users (*N* = 234 million) self-organize into communities called subreddits. These subreddits sometimes reflect a general perspective (such as r/politics or r/philosophy), but more often reflect specific interests (such as r/modeltrains or r/badminton). Unlike Twitter, posts to Reddit are not restricted by length, so the language is less condensed. Reddit dumps for 4 days in the month of November 2017 were downloaded for analysis from (https://files.pushshift.io/reddit/daily/). The language used in Reddit aligns well with the language produced in the structured interviews. Both kinds of language are low in formality, which makes language from Reddit a better fit to the language obtained from the structured interviews than, for example, language from newspaper articles. A second key similarity is that much of the language on Reddit comes from online dialogs. This similarity between Reddit and the structured interviews was imposed by restricting the Reddit corpus to 30,000 individuals who made between 30 and 100 posts in close proximity to one another on the same subreddit forum. In many cases, the multiple posts were made in conversation with other Reddit users. Once the text was cleaned for unusual characters, the main text of the post was abstracted. The Stanford Corenlp Server (https://stanfordnlp.github.io/CoreNLP/corenlp-server.html) was used to process the text, which included sentence tokenization and part-of-speech tagging. The sentences in the text were converted into vectors by summing the word embeddings associated with the words in the sentence. The word embeddings were based on the NYT corpus and were the same as those used in the previous analyses. Once vectorized, the sentences were analyzed for their cosine similarity to the voice cluster vector.

### Evaluation of vector unpacking algorithm

An experiment was conducted to determine whether the semantic densities generated by the vector unpacking algorithm roughly agreed with those of human judges. At the beginning of the experiment, Amazon Mechanical Turk workers (*n* = 59) read an introduction explaining the idea of semantic density, specifically, that semantic density referred to the average amount of information expressed in a set of words. The sentence *Sometimes things are things* was used as an example of a sentence with low semantic density and the sentence *View the latest news* was used as an example of a sentence with high semantic density. Participants were told that they should not base their ratings simply on the number of words in a sentence. For each sentence, participants saw a Likert scale with radio buttons below the numbers 1–10. Above the number 1 was the label “Lowest density” and above the number 10 was the label “Highest density”. To guarantee a range of semantic densities, a stratified sampling approach was used: one third of the sentences had semantic densities (as estimated by the unpacking algorithm) of less than 0.5; one third had semantic densities greater than 0.5 and less than 1, and one third had semantic density equal to 1. Sentence length was controlled for by drawing 12 sentences from sentence lengths of 15, 16, 17, 18, 19, and 20 words. To prevent the rating task from taking too much time, the total number of sentences, *n* = 72, was divided into two rating tasks composed of 36 sentences each. In research involving Amazon Mechanical Turk workers, it is essential to include ratings that can serve as attention checks to protect against workers who are unable or unwilling to complete the task thoughtfully. To this end, participants also rated four attention-check sentences. These sentences were written to be either extremely low (e.g., *I think I thought that but then I didn’t*.) or high (e.g., *My sister’s reaction concerned us*.) in semantic density. Participants were included in the analysis only if they rated the two sentences that were extremely low in semantic density as lower in semantic density than the two sentences that were designed to be extremely high in semantic density. Based on this exclusion criterion, the data from 19 participants were eliminated. Each sentence was rated for its semantic density 15 times. The relation between human and machine estimates of semantic densities was based on an average human judgment for each of the sentences.

## Data Availability

The deidentified results for the training and holdout datasets are available at https://dataverse.harvard.edu/dataset.xhtml?persistentId=doi:10.7910/DVN/K9WKPV

## Code availability

The computer codes used for vector unpacking are available at https://dataverse.harvard.edu/dataset.xhtml?persistentId=doi:10.7910/DVN/MEWSP9
